# Etiologic heterogeneity, pleiotropy, and polygenicity in behaviorally defined intellectual and developmental disabilities

**DOI:** 10.1186/s11689-024-09526-z

**Published:** 2024-03-13

**Authors:** Jessica B. Girault, Olivia J. Veatch, Hyejung Won

**Affiliations:** 1 Department of Psychiatry and Carolina Institute for Developmental Disabilities, University of North Carolina at Chapel Hill, Chapel Hill, USA; 2 Department of Psychiatry and Behavioral Sciences, University of Kansas Medical Center, Kansas City, USA; 3 Department of Genetics and Neuroscience Center, University of North Carolina at Chapel Hill, Chapel Hill, USA

Long before the human genome was sequenced, family studies had established that many neurodevelopmental disorders, including many intellectual and developmental disabilities (IDDs), are heritable and aggregate in families. In the past two decades, large-scale genomics studies have revealed that the genetic architecture of IDDs, and neuropsychiatric disorders (NPDs) more broadly, is incredibly complex. We now appreciate that both rare and common genetic variants interact to influence behavioral and clinical phenotypes in IDDs. This has been eloquently described in work demonstrating that even among individuals with the *same causal genetic mutation* (e.g., 16p11.2 deletion), substantial variability exists in clinical phenotype that can be traced to *other differences in genetic background* [1]. Genome-wide association studies (GWAS) have also demonstrated that IDDs and NPDs are highly polygenic and pleiotropic in nature [2–5], following both a “many-to-one” and “one-to-many” pattern, meaning that many genetic variants contribute to risk for a single diagnosis, and a single genetic variant can influence multiple phenotypes. This genetic evidence has unequivocally demonstrated that behaviorally defined diagnostic classifications used regularly in clinics across the globe are not wholly etiologically distinct, nor completely biologically homogeneous. This is perhaps not surprising, as it echoes decades of evidence documenting familial patterns of broad affectation across IDDs and NPDs (e.g., parent has bipolar disorder, child develops autism spectrum disorder [ASD]). If current diagnostic categories are not adequate to probe underlying biology – which is critical to identifying treatment targets – what then is the path forward?

The articles presented in this special issue of the *Journal of Neurodevelopmental Disorders* tackle this question and offer several important study paradigms and conceptual frameworks that highlight how a *genetics-first* approach to understanding IDDs can shed light on biology and etiology. This special issue contains seven articles from researchers at NICHD-funded Eunice Kennedy Shriver Intellectual and Developmental Disabilities Research Centers (IDDRCs) across the United States and includes conceptual papers on topics ranging from the power of endophenotypes for gene discovery in IDDs (10.1186/s11689-023-09511-y) to the ways in which studying disorders of epigenetic machinery may inform our understanding of typical brain development in IDDs (10.1186/s11689-023-09482-0). Empirical papers in this issue present a variety of strategies to parse etiologic heterogeneity in IDDs by incorporating genetics and measures of brain structure and function (10.1186/s11689-023-09487-9, 10.1186/s11689-023-09498-6), performing cross-diagnostic/disorder comparisons (10.1186/s11689-024-09519-y, 10.1186/s11689-023-09483-z, 10.1186/s11689-023-09487-9), and mining electronic health records (EHRs) linked to biorepositories for genetic clues into a range of psychiatric and co-occurring medical conditions (10.1186/s11689-023-09485-x). These papers exemplify the breadth and depth of the multidisciplinary translational research supported by the IDDRC network and unite around a common theme of incorporating concepts of etiologic heterogeneity, pleiotropy and polygenicity to expand our understanding of both causal mechanisms and intervention targets in IDDs. Here, the editorial team for this special issue synthesizes the body of work from IDDRC investigators to describe two avenues for parsing etiological heterogeneity in IDDs. These paths forward include: [1] detailed phenotyping at multiple scales using genetically informed designs, and [2] harnessing multivariate methodologies and transdiagnostic approaches to reveal novel insights into biological pathways underlying clinical phenotypes.

Many of this year’s collection of articles center around the idea that we can increase our power to understand the biological underpinnings of IDDs by studying phenotypes at multiple levels of analysis along the causal pathway from genes to behavior. Mosconi et al., present a framework for studying “endophenotype trait domains” across “molecular, cellular, circuit, system and behavioral” levels, towards the goal of understanding how genetic variation gives rise to variable clinical phenotypes [6]. This team stresses the importance of focusing on *quantitative outcomes* as opposed to categorical diagnoses, and using family study designs (e.g., twin studies, sibling studies) that allow endophenotypes – which, by definition, must travel in families and track with (sub)clinical traits in both affected and unaffected members – to be empirically evaluated. Mosconi and colleagues also suggest that a developmental approach might be particularly fruitful, as endophenotypes present in early development may be more closely related to causal, and not compensatory, mechanisms. The empirical articles in this issue exemplify the potential utility of this approach. He at al., incorporated multiple levels of analysis to attempt to clarify the pathway from genetic liability for attention deficit hyperactivity disorder (ADHD), to brain structure, to quantitative variation in inattention traits [7]. The authors demonstrated that functionally annotated ADHD polygenic scores (PGS) improved prediction of inattention symptoms compared to the traditional approach that focuses solely on GWAS summary statistics. Age-stratified analyses showed that these effects were developmentally specific and observed primarily in adolescents (ages 12–17 years old) as opposed to children (8–11 years old) and young adults (18–21 years old). He and colleagues also found that functionally annotated ADHD PGS was related to gray matter volumes in the dorsolateral prefrontal cortex (DLPFC), whereas the model testing DLPFC volumes as a mediator of the relationship between PGS and inattention was not significant. This study highlights the needs for large datasets, integrative analysis of multiple phenotypes (e.g., genetic, brain, behavior), and characterization of key traits across development to understand etiological pathways. The findings from Niarchou et al., echoed the importance of a lifespan approach to understanding the pleiotropic genetic architecture of IDDs [8]. They leveraged phenome-wide association studies (PheWAS) in EHR-derived data to detect pleiotropic effects of genomic loci influencing expression of mood disorders and breast cancer by calculating ASD PGS based on GWAS summary statistics. They reported links between ASD PGS and co-occurring clinical concerns that varied in strength based on participant age. In individuals 18 years of age and younger, ASD PGS was predictive of having an autism diagnostic code in medical records, whereas for people ages 26 to 60 years, ASD PGS was predictive of mood disorders and depression. They also found a slight increase in risk for breast cancer later in life in those with higher ASD PGS. In addition to studying associations stratified by age, they also investigated the data split by genetic ancestry. Notably, their findings linking ASD PGS to autism, mood disorder, depression and cancer were only observed when using data obtained from individuals with European ancestry, but not with African ancestry. This finding may reflect the reduced statistical power of the African ancestral dataset (*n* = 12,383 vs. 65,363 with European ancestry), or the limited generalizability of single ancestry GWAS-derived PGS across diverse ancestral populations [9, 10], as the summary statistics used to compute this PGS were largely based on GWAS conducted in individuals with Danish ancestry [11].

Besides PGS which consider cumulative risk attributable to smaller variants, interrogating the accumulation of larger structural variations also appears to be beneficial to pinpointing specific genes of interest in IDDs. For example, Glessner et al., assessed if there was evidence of increased copy number variation (CNV) burden impacting the metabotropic glutamate receptor (mGluR) network – defined as one- or two-degree protein-protein interactors of mGluR1-8 – in individuals with ASD and ADHD [12]. Well-known duplications in specific chromosomal regions that harbor genes within the mGluR network (i.e., 22q11.2, 16p11.2) as well as deletions in two mGluR network genes (i.e., *CNTN4*, *PRLHR*) were enriched in individuals with ASD and ADHD. Beyond the mGluR network, epigenetic factors are also considered key mediators in many neurodevelopmental disorders. Ng and colleagues provide a systematic review of the evidence for alterations in epigenetic machinery that contribute to risk for Mendelian disorders characterized by cognitive dysfunction and behavioral issues [13]. They propose that by comprehensively characterizing similarities and differences in symptomatology across numerous Mendelian Disorders of the epigenetic machinery, as well as focusing on common epigenetic functions of proteins like histone methyltransferases and chromatin remodelers, we can begin to understand the complicated genomic and epigenetic landscape underlying cognitive and behavioral traits associated with IDDs. These studies collectively highlight that directing attention towards specific pathways (e.g., mGluR networks or epigenetic regulators) may have promise in understanding the genetic etiology of IDDs.

The papers by Francisco et al., and Mullin et al., take a different approach to help understand variable expressivity of symptoms in individuals with the same genetically defined syndromes (i.e., fragile X syndrome [FXS], 22q.11.2 deletion syndrome) by comparing behaviorally defined groups or outcomes to delineate developmental trajectories and lend insights into distinct neurobiologies [14, 15]. Mullin and colleagues compare early emerging developmental trajectories among infants with FXS, infants with a family history (FH) of ASD, including infants who later were diagnosed with ASD (FH-ASD) and infants who did not develop ASD (FH-noASD), as well neurotypical infants with the goals of identifying both overlapping and distinct developmental phenotypes. The authors show distinct patterns of nonverbal development in the first year of life in FXS relative to the other infant groups. Further, they document a presymptomatic period in FH-ASD infants characterized by behavioral profiles that were indistinguishable from FH-noASD and neurotypical infants until 12 months. Examining phenotypic variation within a genetically defined population, Francisco and colleagues report that electroencephalography (EEG) can be used to differentiate subgroups of adults with 22q.11.2 deletion syndrome that are characterized by distinct clinical phenotypes. Together these studies demonstrate the utility of combining genetic information (e.g., identified genetic syndromes) with brain and behavioral traits to shed light on the unique developmental pathways and brain mechanisms giving rise to clinical variability.

Another interesting theme that emerged within this multi-scale framework was the important role of sensory and motor behaviors for elucidating neurodevelopmental pathways from genetics to complex clinical phenotypes. Mosconi and colleagues highlight how low-level sensory traits closely model underlying biology and can be assessed at multiple scales and across model systems, making them important targets for future genetic studies [6]. Sensory and motor behaviors may also serve as a foundation for cognition and learning, and thus could have profound cascading effects on brain development and function [16]. The findings from articles led by both Mullin and Francisco lend support for this idea. Mullin and colleagues demonstrated that differences in nonverbal behavior, derived from measures of visual reception and fine motor abilities, differentiated infants with FXS from infants who later developed ASD in the first year of life. Francisco and colleagues found that auditory and visual processing measured by EEG can predict whether individuals with 22q11.2 deletion syndrome will exhibit psychosis. Both studies demonstrate that unique sensory and motor profiles may differentiate individuals with similar diagnostic/clinical features (ASD and FXS) or differentiate outcomes within a single genetic syndrome (22q11.2), thus parsing heterogeneity and providing a potential roadmap to clarifying biological subtypes.

Together the articles in this special issue provide strategies for tackling critical questions about biological mechanisms underlying IDDs. Research teams across the IDDRCs are charting a course forward, where behaviorally defined IDDs can – *and should* – be reconceptualized amid mounting evidence of shared polygenic risk and pleiotropic genetic effects. They highlight the power of family study designs and transdiagnostic and cross-disorder research at multiple scales to further our understanding of the pathway from genes to brain, from brain to behavior, and from behavior to clinical phenotype. This framework holds great promise for informing the next generation of research into targeted treatments for IDDs based on biological subtype.
